# Exploring molecular reorientations in amorphous and recrystallized felodipine at the microscopic level†

**DOI:** 10.1039/d0ra07266d

**Published:** 2020-10-08

**Authors:** A. Pajzderska, J. Jenczyk, J. P. Embs, J. Wąsicki

**Affiliations:** Faculty of Physics, Adam Mickiewicz University Uniwersytetu Poznańskiego 2 61-614 Poznań Poland apajzder@amu.edu.pl; NanoBioMedical Centre, Adam Mickiewicz University Wszechnicy Piastowskiej 3 61-614 Poznań Poland; Laboratory for Neutron Scattering and Imaging, Paul Scherrer Institut 5232 Villigen Switzerland

## Abstract

Molecular reorientations were studied in amorphous, partially and fully recrystallized felodipine (calcium channel blocker, a drug from the family of 1′,4-dihydropyridine) using a set of experimental methods: high-resolution solid-state nuclear magnetic resonance (NMR), relaxometry NMR and quasielastic neutron scattering (QENS). The results were compared with molecular dynamics in crystalline felodipine previously investigated [A. Pajzderska, K. Drużbicki, M. A. Gonzalez, J. Jenczyk, J. Mielcarek, J. Wąsicki, Diversity of Methyl Group Dynamics in Felodipine: a DFT Supported NMR and Neutron Scattering Study, *CrystEngComm*, 2018, **20**, 7371–7385]. The kinetics of the recrystallization was also studied. The most stable sample was the sample stored in a closed ampoule (at room temperature, in 0% RH) and its complete recrystallization lasted 105 days. In the fully recrystallized sample, the same molecular reorientation identified in the crystalline form was detected, so reorientations of all methyl groups and the ethyl ester fragment. In the partially recrystallized sample, static disorder caused by the two positions of both side chains was revealed. In the amorphous sample the reorientation of all methyl groups was analyzed and the distribution of correlation times and energy barriers connected with the loss of long-range ordering and disorder of side chains were analyzed. Additionally, inhibition of reorientation in the ethyl ester fragment was observed.

## Abstract 2

Molecular reorientations were studied in amorphous, partially and fully recrystallized felodipine using a set of experimental methods: high-resolution solid-state nuclear magnetic resonance (NMR), relaxometry NMR and quasielastic neutron scattering (QENS).

## Graphical abstract

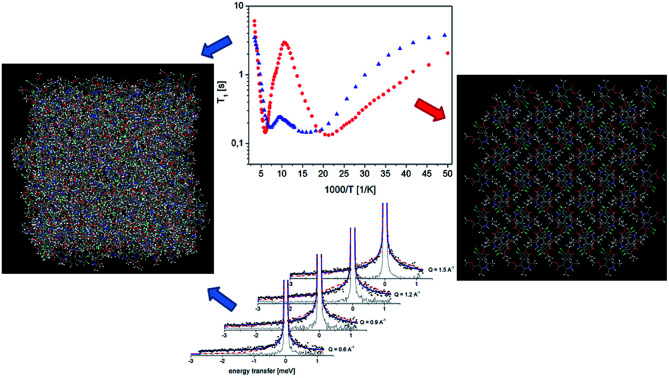

## 1 Introduction

The bioavailability of active pharmaceutical ingredients, which also determines the effectiveness of therapy, is directly related to both the solubility and dissolution rate. Substances in an amorphous state usually have better solubility compared to crystalline forms, which means greater therapeutic efficacy at lower doses, thus reducing the risk of side effects. On the other hand, the use of amorphous substances is associated with problems resulting from their low physicochemical stability. Amorphous substances are thermodynamically unstable and tend to crystallize, which in the case of medicinal substances, can lead to serious, unexpected pharmaceutical and therapeutic consequences. Therefore, at present, understanding the properties of amorphous systems and the processes of recrystallization of these systems is a matter of interest and intensive research.

The physicochemical properties of organic systems, including amorphous ones are also influenced by molecular dynamics, and it is believed that mobility is one of the main factors that govern their physical stability,^1,2^ therefore analysing this seems to be an important factor of the characteristics of amorphous samples. Currently, it is widely believed that knowledge of the reorientation and mobility of molecular amorphous systems may be the key to understanding their physicochemical properties and an indication of effective methods of increasing physical stability. The rate of structural relaxation (α relaxation) close to the glass transition correlates with a high tendency to crystallize pharmacologically active ingredients.^3–5^ In addition, molecular reorientations described by β relaxation^3,5,6^ can influence the recrystallization of amorphous systems. Information on molecular mobility is obtained mainly on the basis of broadband dielectric spectroscopy (BDS). Nuclear magnetic resonance^7–10^ and quasielastic neutron scattering methods are also used to analyse molecular reorientation in amorphous systems.^11,12^

Moreover, the macroscopic properties of amorphous substances could depend on the dynamics of molecules and molecular fragments.^8^ Thus, understanding the dynamics of molecular groups, including methyl groups, is of general interest in drug design. Reorientations of molecular groups (methyl, ethyl and larger fragments of molecules) depend on intra- and intermolecular interactions. As is known, in crystalline systems, the latter depend on long-range ordering, while in amorphous systems, they depend on short-range ordering. Therefore, comparing molecular reorientations of substances in crystalline and amorphous forms provides information on existing interactions. This information is especially important if the molecules do not change conformation when switching from a crystalline to an amorphous form. In such cases, it can be assumed that the intramolecular interactions will not change or any changes will be very slight. For this reason, the reorientation analysis of molecular groups modulated by short-range inter-molecular interaction is a sensitive probe that provides information on this type of interaction.

The aim of this work is to develop a microscopic model of molecular reorientation for amorphous felodipine (FLD), to investigate the process of its return to a crystalline form (recrystallization) in a wide temperature range below *T*_g_ (glass temperature) and to study the dynamics of the partially and fully recrystallized form of felodipine. Molecular reorientation will be investigated using ^1^H NMR (nuclear magnetic resonance), ^13^C CP-MAS NMR and QENS methods for temperatures below *T*_g_, while the recrystallization process will be monitored by the NMR relaxation method.^13^

**Fig. 1:**
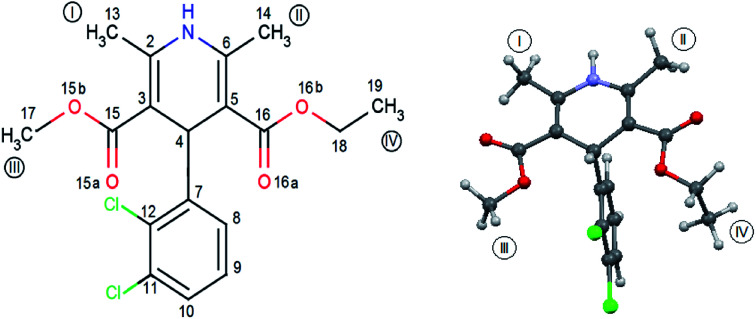
Molecular geometry of felodipine along with the methyl groups and atom notation adopted throughout this work. On the right – the conformation of felodipine molecule corresponding to polymorph I.^39^

FLD is the subject of interest of many researchers^16–34^ and can be treated as a model system, but so far little is known about molecular reorientations in the amorphous form of FLD. Interest was focused mainly on the study of various aspects of the stabilization of the amorphous form of FLD^19,22–24,35,36^ and recrystallization processes.^18,20,21,25–33^ The aforementioned studies were performed mostly at the macroscopic level. The exceptions are papers,^14,15^ in which the authors analyzed how the amorphization of a series of dihydropyridines (including FLD) influences intermolecular hydrogen bonds, using optical spectroscopy (FTIR and Raman) methods. Felodipine is a important dihydropyridine calcium antagonist, frequently prescribed as an antihypertensive agent,^37^ and it is a good example of an orally distributed pharmaceutical solid suffering from poor bioavailability,^38^ due to peculiarly low water solubility, *i.e.* 0.5 mg l^−1^. FLD is a relatively rigid molecule with limited conformational freedom (Fig. 1), emerging as an attractive model to study bulk amorphous and recrystallization properties.

Recently, we have described molecular reorientations occurring in crystalline FLD^13^ in detail by means of spectroscopic (nuclear magnetic resonance – NMR and quasielastic neutron scattering – QENS) and computational (periodic density functional theory – plane-wave DFT) methods. In this work, the same spectroscopic methods were used to study molecular reorientation in amorphous FLD. Comparison of molecular reorientations occurring in amorphous and crystalline FLD will allow an assessment of how loss of crystal long-range order will affect positional as well as orientational local ordering.

## 2 Experimental details

### 2.1. Sample

The polycrystalline sample of FLD was characterized in a previous paper,^13^ and the recorded diffraction spectrum confirmed that the sample corresponds to the most stable polymorph I.^39^

For NMR measurements, polycrystalline FLD was placed in a glass ampoule with a diameter of 7 mm and a length of 90 mm, outgassed and sealed off. Then the ampoule was heated up to 425 K (about 10° above melting point) and kept at that temperature for about 15 minutes. Then the sample was cooled to 100 K with liquid nitrogen for about 20 minutes (giving an average cooling rate of ∼16 K min^−1^). After heating the sample to room temperature, the FLD was transparent and of a slightly yellow color. Four bulk samples were prepared as described above, each of them with a mass of ∼0.3 g. Three of these were opened and placed in exsiccators with KI, MgCl_2_ and NaCl giving a relative humidity (RH) of 32.8%, 68.9% and 75.3% respectively. The RH of the sealed sample was 0%. All samples were stored at 303 K.

Similarly, an amorphous FLD was also prepared in a zirconium rotor with a diameter of 4 mm (for measuring ^13^C NMR spectra) and a cylindrical aluminum container for QENS measurements. The measurements were carried out immediately after amorphization.

### 2.2. Experimental methods

^1^H NMR spin–lattice relaxation times (*T*_1_) were measured using the saturation-recovery method with a ^1^H NMR pulse spectrometer working at a resonant frequency of 25.0 MHz (El-Lab Tel-atomic Co.). The measurements were performed in the range from 20–298 K and the temperature was stabilized with an accuracy of 0.1° by a helium–nitrogen cryostat.

Solid state NMR spectra were acquired on a 400 MHz Agilent spectrometer equipped with a Wide Bore Triple Resonance T3 MAS XY probe. ^13^C detection was realized using a Cross-Polarization (CP), Magic Angle Spinning (MAS) experiment with dipolar decoupling of protons during acquisition. The samples were placed into 4 mm diameter zirconia rotors and spun at 5 kHz frequency. 4096 transients were accumulated. Repetition delay was 10 s. Spin–lattice relaxation times *T*_1_ for ^13^C were estimated using a two-pulse sequence for magnetization recovery observation with the repetition time set to 125 s.

Quasielastic neutron scattering (QENS) measurements of crystalline and amorphous felodipine were performed on a FOCUS (PSI, SINQ) time-of-flight spectrometer. The sample was placed in a specially designed container consisting of a hermetically sealed aluminum hollow cylinder (mass of sample = 1.4 g). QENS spectra were collected in the Q-range 0.3–1.8 Å^−1^ using a PG002 monochromator, giving an incoming neutron wavelength *λ*_0_ = 5.75 Å and an energy resolution equal at ∼0.04 meV (FWHM). This means that this spectrometer probes motions in the ps timescales. First, the crystalline sample was measured at 300 K. Then, as was mentioned above in Section 2.1, it was heated up to 430 K (10 K above phase transition) and an amorphous sample was prepared by the melt-quench cooling method and also measured at 300 K. Powder diffraction spectra (which are measured simultaneously on FOCUS) allowed the phase transitions to be followed, and the disappearance of Bragg peaks confirmed the amorphisation of the sample. The sample holder was weighed before and after the measurements to confirm that it was hermetic and no mass losses were detected. Additionally, an empty container and a vanadium sample were measured at room temperature to be used in the background and normalization procedures, respectively.

Raw data were treated with the DAVE package,^40^ which performs the standard corrections, including background subtraction and self-absorption correction. Then, using a vanadium spectrum as an elastic scattering standard, the intensities were normalised in order to correct for different detector efficiencies. Finally, detectors where Bragg peaks influenced the collected spectra of crystalline felodipine were removed from further analysis in order to focus only on the incoherent quasielastic neutron scattering. The spectra were fitted using the STR_FIT program in Lamp,^41^ which enables numerical convolution with the instrumental resolution function *R*(*Q*,*ω*) determined from a measurement of a vanadium standard sample.

## 3 Results

### 3.1. Recrystallization time

To produce a description of molecular reorientation over a wide temperature range in amorphous FLD, this sample should undergo full recrystallization in at least two orders of magnitude longer than the time needed for measurements. This time depends on the measurement method, but the minimum is 4 days. Therefore, the first stage of the study was to determine the time after which the full recrystallization of four amorphous FLD samples prepared in the way described above took place (2.1 Sample). To determine this time, a method we had developed based on NMR relaxation was used.^42^Table 1 summarizes the results obtained for four samples and, additionally, the results previously obtained for samples with grain sizes of 0.1 mm and 1 mm.^42^

**Table 1:** Time after which complete recrystallization of the FLD amorphous sample took place

Type of sample	Relative humidity [%]	Time [days]
Bulk-open	75.3 (NaCl)	15
Bulk-open	68.9 (KI)	17
Bulk-open	32.8 (MgCl_2_)	22
Bulk-close	0	105
Grain size of 1 mm (ref. 42)-open	30	4.5
Grain size of 0.1 mm (ref. 42)-open	30	0.8

Analysis of the data contained in Table 1 shows that the time after which the amorphous FLD fully recrystallizes depends greatly on its grain size and relative humidity. The bulk amorphous sample in a closed ampoule had the longest recrystallization time and therefore this sample was chosen for further research.

### 3.2. The identification of molecular reorientations in amorphous felodipine

The internal molecular mobility of crystalline FLD is associated with the reorientational dynamics of four methyl groups, accompanied by the reorientation of the ethyl fragment around the O_16b_–C_18_ axis.^13^ Computationally-supported relaxation NMR experiments revealed that methyl group no. I–III reorients across low activation barriers, while methyl group no. IV has a high activation energy. Our experiment results show that CH_3_ no. I and II undergo quantum rotational tunneling, which is also observed for a methyl ester part (CH_3_ no. III). It is worth underlining the frequencies of reorientation (and the related correlation time *τ*_c_) of these methyl groups are strictly separated. It is interesting how the amorphization of the sample influences these reorientations.

#### 3.2.1. ^13^C CP MAS NMR

**Fig. 2:**
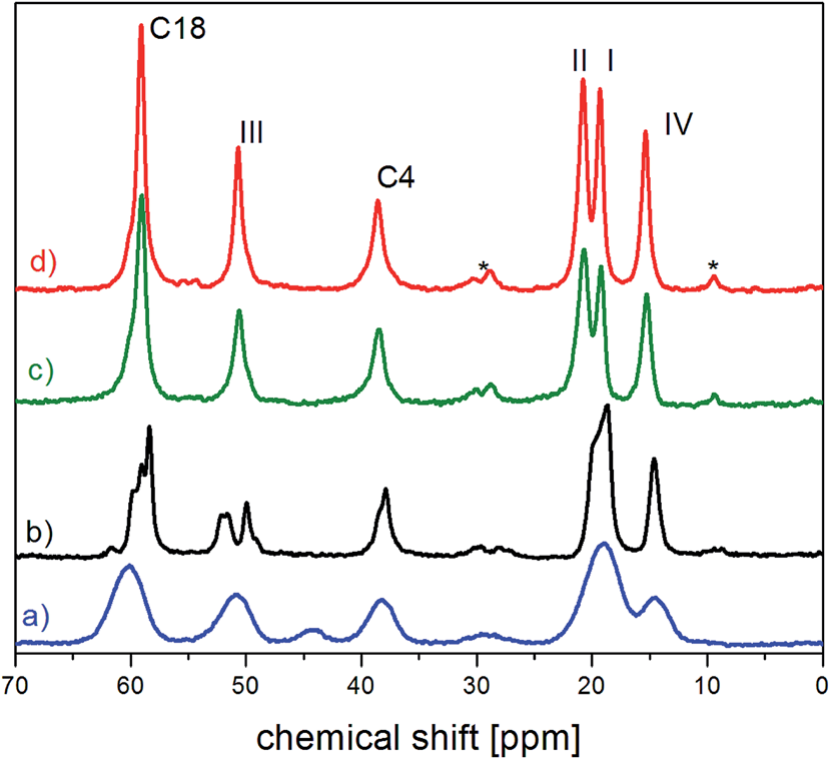
The fragment of ^13^C CP MAS spectra of (a) amorphous (b) partially recrystallized (c) fully recrystallized (d) crystalline FLD.^13^ The asterisks denote satellites.

In the first stage of the study, ^13^C CP MAS spectrum for the amorphous sample will be analyzed. Fig. 2a displays the spectrum together with the previously detected one for the crystalline sample (Fig. 2d) covering the response of the terminal groups,^13^ while the entire spectra are presented in ESI (Fig. 1S†).

For the amorphous sample, compared to the crystalline one, we observed a significant widening of all lines and merging of two peaks related to carbon atoms of methyl groups I and II. Their width is approximately 4 times greater than for a polycrystalline sample and is mainly caused by the modification of intra and intermolecular interactions.^1^ Modification of intermolecular interactions leads to a loss of translational symmetry and to the disappearance of long-range ordering. This strongly indicates that the side chains (methyl ester COOCH_3_ and ethyl ester COOCH_2_CH_3_) are also at least partially disordered.

**Fig. 3:**
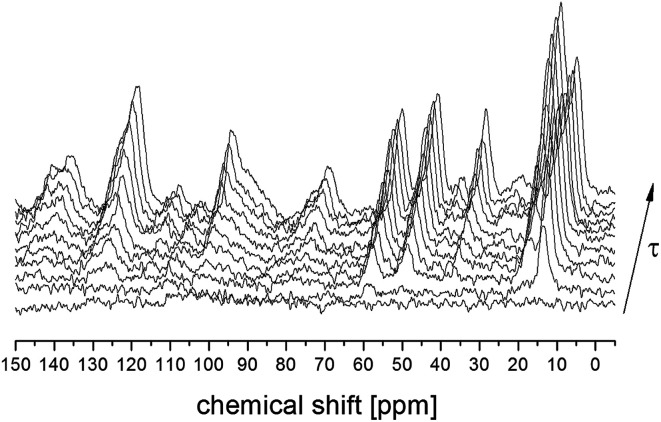
^13^C CP MAS NMR spectra for amorphous FLD in a function of time *τ* between radio frequency pulses.

*T*_1_ relaxation time for ^13^C atoms was also measured for all the lines analyzed above. Fig. 3 shows the ^13^C CP MAS spectra recorded for different time intervals *τ* between radio-pulses for the amorphous sample. The single-exponential recovery of magnetization was observed and could be described by the formula:

1 *M*_z_ = *M*_0_(1 − exp(−*t*/*T*_1_))

where *T*_1_ is the time of relaxation, *M*_0_ is the magnetization in the state of thermodynamic equilibrium.

The obtained values of *T*_1_ are given in Table 2 together with the relaxation times previously measured for the crystal sample.^13^ The experiment allowed us to extract the spin–lattice relaxation times for each individual terminal carbon, however the *T*_1_ relaxation times for 1,4-dihydropyridinium and chlorophenyl rings carbons are much longer, with values comparable to the repetition time. Nevertheless, we can estimate that their values do not change after the amorphisation of the sample.

**Table 2:** Chemical shifts and *T*_1_ relaxation time obtained from ^13^C CP MAS spectra for amorphous, partially recrystallized and crystalline^13^ FLD

Carbon	Crystalline^13^		Partially recrystallized		Amorphous	
	*δ* [ppm]	*T*_1_ [s]	*δ* [ppm]	*T*_1_ [s]	*δ* [ppm]	*T*_1_ [s]
C19 IV	14.8	0.6	14.7	0.6	14.4	1.4
C13 I	18.7	39.0	18.6	37.0; 22.5	19.1	22.5
C14 II	20.1	40.0	19.9			
C17 III	50.1	24.0	50.0; 51.9	24.5; 14.3	50.9	14.3
C18 CH_2_	58.5	32.0	58.4; 59.2; 60.4	32.1; 12.2	60.4	12.2

The values of the relaxation times *T*_1_ in amorphous FLD were reduced two- or even threefold (for the carbon atom of the CH_2_ group) in comparison to crystalline sample, suggesting higher local mobility of FLD. The exception is carbon from methyl group IV, for which the relaxation time increased from 0.6 s to 1.4 s. The longer *T*_1_ time for C_19_ from the IV methyl group indicated that the reorientation of the IV methyl group around the O_16b_–C_18_ axis (which means the reorientation of ethyl fragment) was inhibited. Our previous calculations^13^ of a two-dimensional potential energy scan (PES) of the energy dependence between the orientation of methyl group no. IV and the reorientation of the ethyl ester fragment showed that the local minimum on the PES was directly associated with the reorientation of the side ethyl fragment. Since the results of ^13^C NMR indicated a slowing of this reorientation, we can assume that as a result of amorphization, the PES changed, and this ethyl fragment is in a deeper, local minimum, hence its reorientation can only occur through a higher barrier.

#### 3.2.2. ^1^H NMR spin-relaxation time *T*_1_

**Fig. 4:**
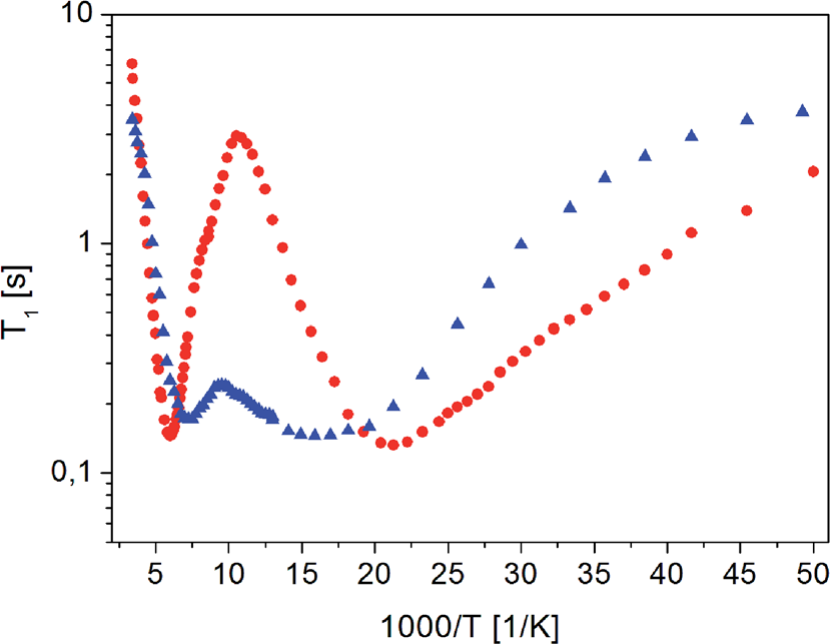
The dependence of relaxation time *T*_1_ on the inverse of temperature for (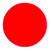) crystalline^13^ and (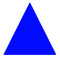) amorphous FLD.

To better understand molecular reorientation, measurements of the spin–lattice relaxation time *T*_1_ in a wide temperature range were made immediately after amorphization, and the results obtained are shown in Fig. 4.

For both the crystalline and amorphous samples in the whole temperature range, a single-exponential recovery of magnetization was observed and could be described by the formula (1). For the amorphous sample the ln *T*_1_(10^3^/*T*) relationship shows two clearly outlined minima: a high temperature at 142 K and a low temperature at 63 K, although a shallow minimum is not observed. The low-temperature minimum of relaxation time *T*_1_ is shifted towards high temperatures compared to the low-temperature minimum previously recorded for the crystalline sample. On the other hand, the high-temperature minimum is observed at lower temperatures than for the crystalline sample. At the lowest temperatures, the relation of ln *T*_1_(10^3^/*T*) is clearly non-linear, and the relaxation times *T*_1_ slightly depend on the temperature.

The detection of a minimum spin–lattice relaxation time *T*_1_ means that in the sample studied there is a molecular reorientation with a frequency comparable to the resonance frequency (the condition for the occurrence of a minimum *T*_1_ can be written as follows: *ω*_0_*τ*_c_ = 0.616, where *ω*_0_ is the resonance frequency and *τ*_c_ is the correlation time).

The shift towards high temperatures of the low-temperature minimum (which reflects reorientations of methyl groups I, II and III) means that the reorientations of these methyl groups are slowed down, because the correlation time *τ*_c_ increases. This also means that, according to the Arrhenius equation, these reorientations occur through higher potential energy barriers compared to the crystalline sample. However, for reorientation of the IV methyl group (high-temperature minimum of *T*_1_), the energy barrier decreases compared to its height for the crystalline sample. These considerations are only of a qualitative nature. To quantify these results, the *T*_1_ relaxation time minima were described using classical BPP equations:2
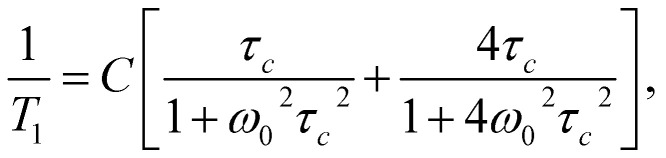

3 *τ*_c_ = *τ*_0_ exp(*E*_A_/RT),

where *ω*_0_– Larmor frequency, *C* - constant, *τ*_c_ - correlation time and *E*_A_ is energy barrier.

**Fig. 5:**
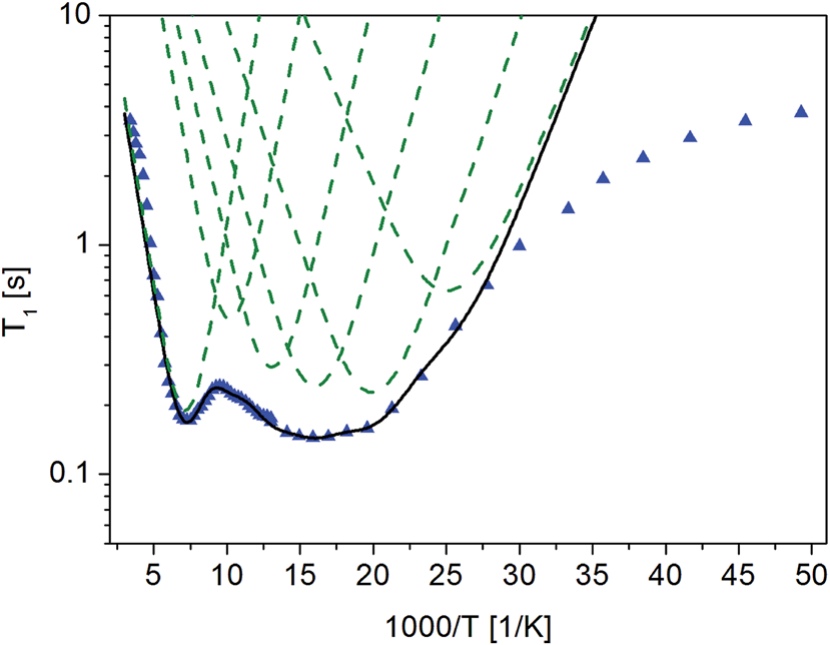
Dependence of relaxation time *T*_1_ on the reciprocal temperature for amorphous FLD. Measured values are marked with points (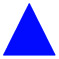), best fitting to experimental values are marked with continuous solid line (black) and individual BPPs processes are marked with dashed lines (green).

The best fit to the experimental results was obtained when the high-temperature minimum was described by one BPP process and the low-temperature minimum by the combination of five such processes. Fig. 5 shows the experimentally determined relationship ln *T*_1_(103/*T*), individual relaxation processes and their sum (solid line), while the activation energies are collected in Table 2.

The description of the low-temperature minimum using five relaxation processes should be commented. For the crystal sample, this minimum refers to reorientation of three methyl groups (I, II, III). Therefore, if the reorientations of three methyl groups in an amorphous sample are described by five, or perhaps more relaxation processes, it means that there is a distribution of the height of energy barriers of these reorientations. This distribution is also indicated by the fact that the values obtained for the activation energies vary over a wide range, from 3.0 to 6.8 kJ mol^−1^. The reason for this distribution of barrier heights in an amorphous system is the different environment of FLD molecules, due to the lack of long-range ordering suggested by the ^13^C NMR study. It should be remembered here that for methyl groups I, II and III in the crystalline sample, tunneling at low temperatures is detected, while in the amorphous sample, this process is not observed.

As the shallow minimum related to reorientation of the ethyl fragment around the axis determined by the O_16b_–C_18_ bond is not detected in the temperature range measured, it should be assumed that this motion of the side chain is slowed down drastically. This observation is in agreement with the results of ^13^C NMR data discussed in the previous section, which also indicated a slowing of this reorientation. Therefore, this ethyl fragment is located in the deeper potential energy minimum, but at the same time, the lack of long-range ordering causes an almost two-fold decrease in the height of the energy barrier for reorientation of the methyl group IV located at the end of this side chain.

The proposed model of reorientation of methyl groups in the amorphous sample describes the experimental results well, except for those at the lowest temperatures. As mentioned before, the measured values of T_1_ times are shorter than the values determined on the basis of the proposed model. In the area of low temperatures, when the reorientations of methyl groups are already “frozen” (that is, they occur at a very low frequency), other processes, for example, proton jumps in hydrogen bridges, may influence T_1_ relaxation time.^43,44^ There are two types, relatively weak, N–H⋯O intermolecular hydrogen bonds with lengths N⋯O 3.155 A and 3.237 A in crystalline felodipine.^39^ The FTIR and Raman spectroscopy methods indicated that in the amorphous sample, the hydrogen bond lengths change and their strength is greater than in the crystalline sample.^14^ Therefore, we can say that at the lowest temperatures, relaxation can be dominated by the classical dynamics of protons in hydrogen bridges.

#### 3.2.3. Quasielastic neutron scattering

The next stage of the research was the study of quasi-elastic neutron scattering to verify whether the distribution of correlation times would be observed in the amorphous sample, and to observe this distribution by independent method.

First, the QENS spectra of crystalline FLD were analyzed. As we know from a previous study,^13^ methyl group reorientations are characterized by different activation energies and correlation times. Previously performed QENS measurements for crystalline FLD on a time-of-flight spectrometer (IN6, Grenoble, ILL) and DFT calculations showed that the reorientations of two methyl groups (no. I and II) at room temperature are characterized by correlation times of ps. Therefore, it should also be assumed that the spectra recorded on the FOCUS spectrometer (with the resolution comparable to IN6) will reflect the orientations of these methyl groups. The spectra were fitted assuming the reorientation of methyl groups using this specific model corresponding to jumps between three equidistant sites on a circle of radius *r* (ref. 45) by the following expression (convoluted with the resolution function *R*(*Q*,*ω*)):4
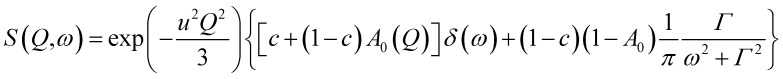
where; *δ*(*ω*) is the delta function, *A*_0_(*Q*) is the elastic incoherent structure factor (EISF), 
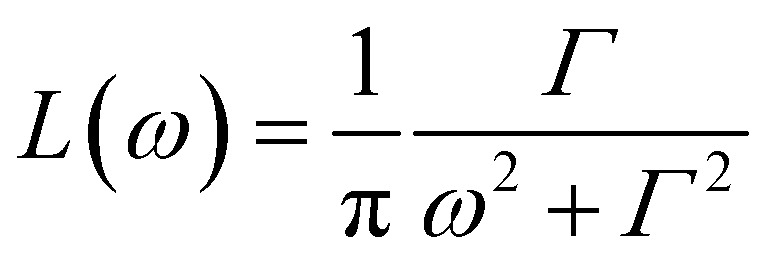
 is a Lorentzian function of half-width at half-maximum *Γ*, u^2^ - the Debye–Waller factor, *Q*-dependence of *A*_0_(*Q*) is given by:5
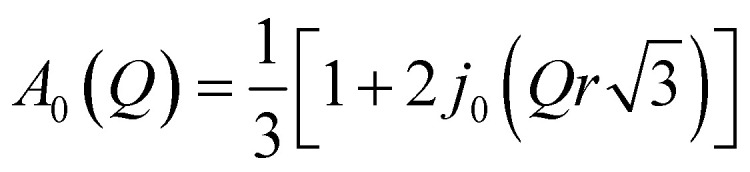


In general, all atoms in the sample could contribute to the total scattering, but due to a large incoherent scattering cross-section of hydrogens, their contribution represents more than 90% of the total scattering and the contribution from other atoms (nitrogen, carbon, oxygen, and chlorine) can be neglected. Thus, *c* describes the fraction of immobile hydrogen atoms in the experimental time scale.

**Fig. 6:**
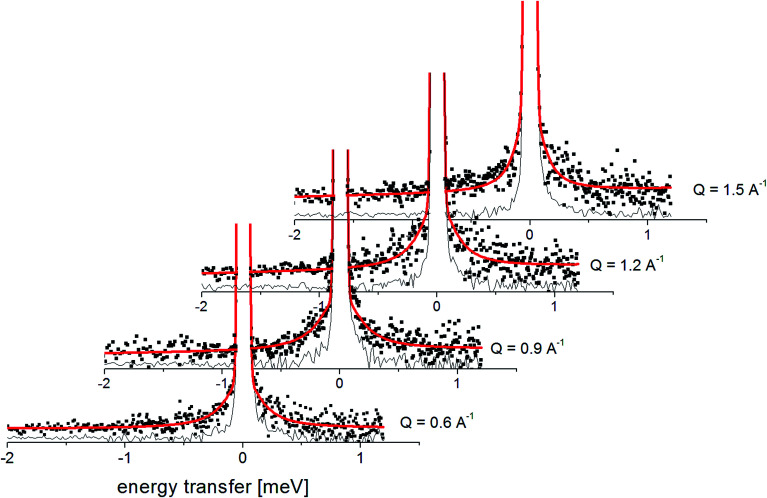
The experimental QENS spectra (points) for crystalline FLD at 300 K. The solid red line shows the fits given by eqn (4), the black line shows the elastic component.

This model describes the experimental data very well, as is shown in Fig. 6, and the fitted *c* parameter indicates that (as we expected) the motion observed in the QENS experiments is effectively the rotation of two methyl groups between three equivalent positions. As the correlation time *τ*_c_ is inversely proportional to the half-width of the Lorentz function *Γ*, we can also say that the *τ*_c_ of this motion at room temperature is 1.1 ± 0.1 ps.

The analyzed spectra for crystalline FLD are best fitted by the function containing one Lorentz function describing the quasielastic broadening. This means that both groups CH_3_ are characterized by the same correlation time. However, the spectra of the amorphous sample are not described well by the eqn (4), even with the assumption that two Lorentzian functions can describe the quasielastic contribution. This can be understood by referring to earlier studies,^11,46^ and our ^1^H NMR data discussed above, which indicate that the reorientation of methyl groups in the amorphous phase cannot be described by a single correlation time. It means that the quasielastic broadening could not be described by a function containing a single Lorentz function. Instead, we find that the description of the experimental curves requires the use of several Lorentz functions. Therefore, the following model was chosen to describe the experimental results:6
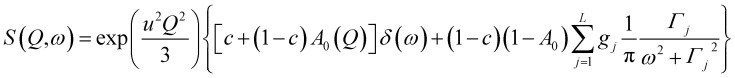
where:
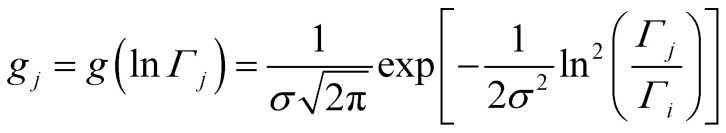


This model is based on the rotation distribution model, which considers a distribution of jumping rates^47^ satisfactorily applied to amorphous diazepam.^11^ For each jumping distance, instead of a single *Γ*_i_ value, a distribution of HWHMs is used. The distribution is represented by *L* values of the HWHM (*Γ*_j_) with associated weights *g*_j_ taken from a log-normal distribution of standard deviation *s* and normalized such that 
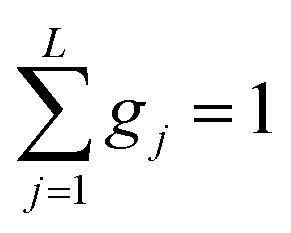
. The *Γ*_j_ are chosen equally spaced on a logarithmic scale in the range 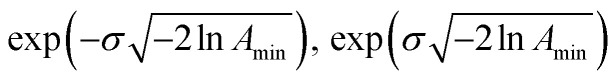, where *A*_min_ is the cut-off chosen for the value of the distribution function with respect to its maximum. Here we used *L* = 21 and *A*_min_ = 0.1.

**Fig. 7:**
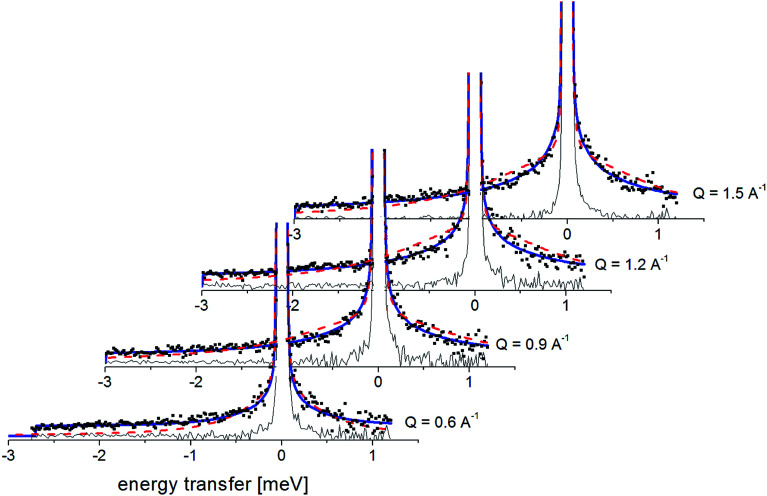
The experimental QENS spectra (points) for amorphous FLD at 300 K The solid lines correspond to the fits obtained using eqn (6), while the dashed lines correspond to those using eqn (4). The difference between both fits shows clearly that the model assuming only one correlation time (one single Lorentzian function) cannot describe the experimental data well. The black line shows the elastic component.

**Fig. 8:**
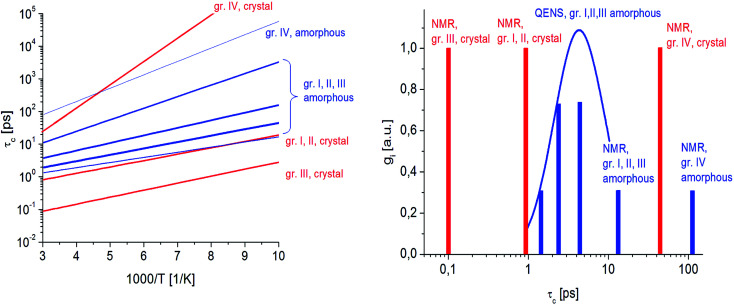
(Left) Dependence of correlation times *τ*_c_ on the reciprocal temperature for reorientation of methyl groups in crystalline and amorphous FLD. (Right) distribution of correlation times for amorphous FLD (blue line) obtained from the fit of QENS data at 300 K for methyl groups along with the values of *τ*_c_ obtained from NMR data for crystalline (red columns) and amorphous samples (blue columns).

Fig. 7 presents the QENS spectra of amorphous FLD recorded at 300 K and several Q values together with the results of the fits obtained with eqn (6), which describes the experimental data very well. As mentioned above, the half-width *Γ*_j_ obtained from the fitting of the QENS spectra is directly related to the correlation time *τ*_c._ As each Lorentz function of width *Γ*_j_ represents a contribution described by the weight *g*_j_, it is possible to obtain a distribution of correlation times. These are shown in Fig. 8 along with the temperature dependence of correlation times that were obtained from the previously discussed NMR results based on activation parameters and Arrhenius dependencies.

In this figure, red lines indicate relaxation processes for the crystalline sample, and blue lines for the amorphous sample. The line thicknesses are proportional to the number of reorientating methyl groups (Fig. 8 left). In crystalline felodipine, the frequencies of reorientation of group III and groups I and II are clearly separated, but we see that in the amorphous sample, the distribution of correlation times of methyl groups I, II and III at 100 K covers at least two orders of magnitude and decreases with its increase to one order size at room temperature. We can also notice that in the temperature range studied, the reorientations of groups I, II and III in the amorphous sample are slower than in the crystalline sample. The distribution of correlation times extracted from QENS spectra is complementary to NMR data and shows that the correlation times at room temperature for reorientation of groups I, II and III are in the range of 1 to 10 ps, while the maximum distribution is 4 ps.

### 3.3. The kinetics of recrystallization

The next stage of the research involved measurement of relaxation times *T*_1_ for the amorphous sample performed in the temperature range from 300 K to 77 K as a function of the time that had elapsed from the amorphization of the sample. A week after the sample was amorphized, the recovery of magnetization was non-exponential. Similarly, non-exponential recovery was observed for a further 13 measurements, in particular in the temperature range from 167 K to 77 K. All the results of magnetization measurements could be described with a good approximation by the following formula:

7 *M*_z_ = *M*_0_^1^(1 − exp(−*t*/*T*_1_^1^)) + *M*_0_^2^(1 − exp(−*t*/*T*_1_^2^))

where:8
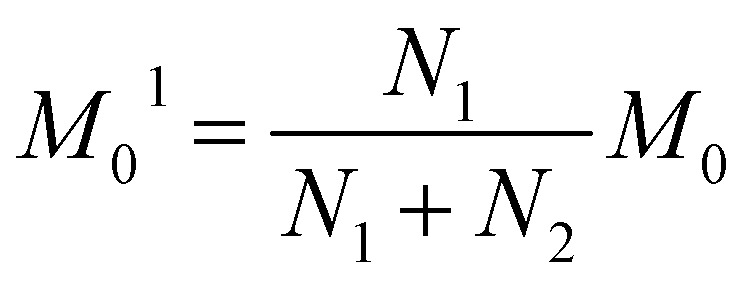
9
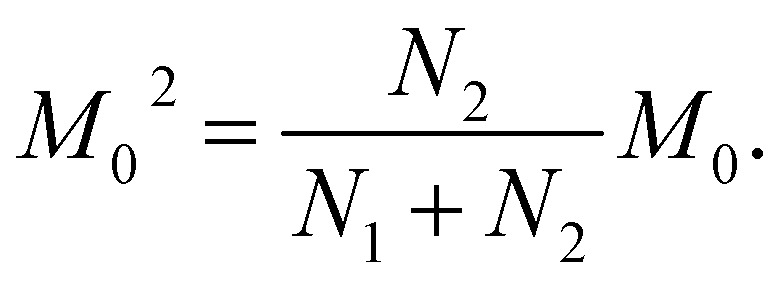


**Fig. 9:**
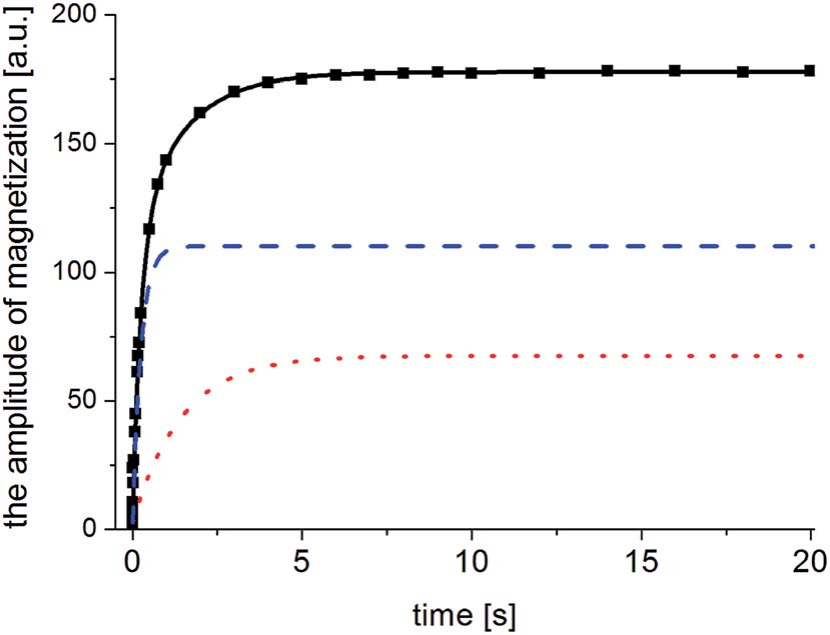
Magnetization *M*_z_ (points) *versus* the time distance between the saturating series and probing pulse (*τ*) recorded for the FLD sample at 300 K after 38 days of amorphization. The magnetization of the amorphous phase is marked by a blue, dashed line and that of the crystalline phase by a red, dotted line. The resulting magnetization in a two-phase system is marked by a black, solid line.

The two components in formula (7) mean that in the sample studied, there are two nuclear spin systems *N*_1_ and *N*_1_, which relax with times *T*_1_^1^ and *T*_1_^2^, respectively, and their contribution to total magnetization is *M*_0_^1^ and *M*_0_^2^. An example of the magnetization recovery is shown in Fig. 9.

**Fig. 10:**
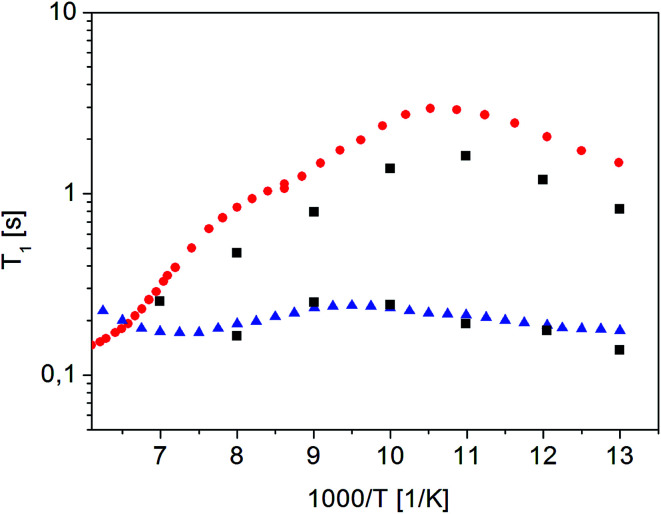
Dependence of relaxation time *T*_1_^1^ and *T*_1_^2^ on the reciprocal temperature on the 38th day after sample amorphization (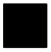). The times *T*_1_^1^ and *T*_1_^2^ obtained for the other measurements as a function of time since amorphization have the same values, but differ in amplitude (magnetization value). Crystalline^42^ (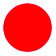) and (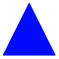) amorphous sample immediately after its amorphization

Fig. 10 shows the inverse temperature dependence of relaxation times *T*_1_^1^ (with shorter values) and *T*_1_^2^ (with longer values) determined from formula (7) in the temperature range from 167 K to 77 K on the 38^th^ day after sample amorphization. This figure also shows the results of measurements for the crystalline and amorphous sample (immediately after its amorphization) for comparison purposes.

It is clear that the temperature dependence of the *T*_1_^1^ relaxation time component covers the *T*_1_ relaxation time values for the amorphous sample. The temperature dependence of *T*_1_^2^ relaxation time, on the other hand, is very similar to the dependence of *T*_1_ relaxation time for a crystalline sample, although the *T*_1_^2^ time values are slightly shorter than the *T*_1_ value for the crystalline sample. It should be underlined that the relaxation time values *T*_1_^1^ and *T*_1_^2^ do not depend on the time elapsed since the sample was amorphized (at the limit of the measurement error).

**Fig. 11:**
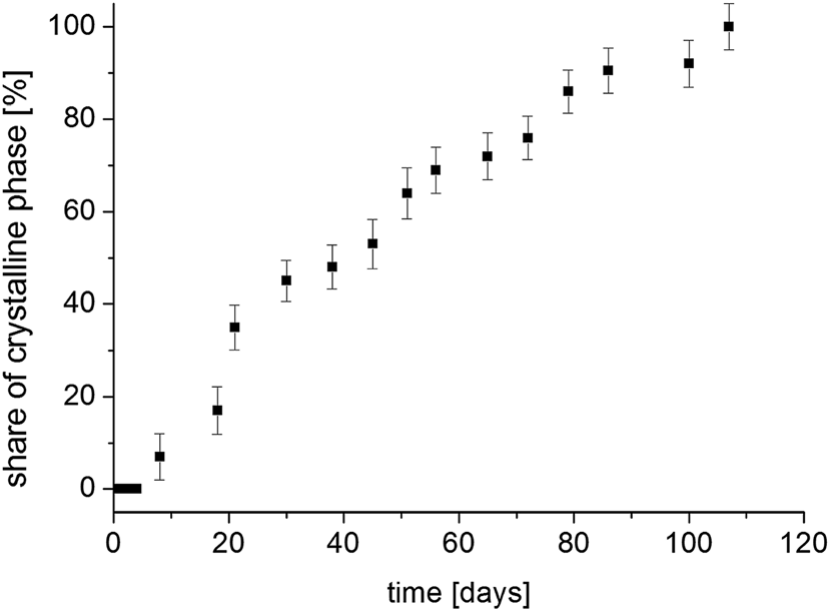
The contribution of the crystalline phase in FLD sample as a function of the time from the sample amorphization.

The situation is different with the magnetization components *M*_0_^1^ and *M*_0_^2^, whose values strongly depend on the time elapsed since the sample was amorphized. If the recrystallization process begins in a homogeneous amorphous sample, it means that a two-phase system is formed and the sample becomes heterogeneous. If the values of *T*_1_ spin–lattice relaxation times (which are the time constant at which nuclear spins exchange their energy with the lattice) for the amorphous and crystalline systems are different and when there is a slow spin exchange between them, nuclear magnetization *M*_z_ will recover in such a sample two-exponentially and it can be described by the formula (7), and the values of amplitudes *M*_0_^1^ and *M*_0_^2^ are proportional to the number of nuclear spins (in our case, protons) that are in individual phases. Therefore, the amplitudes *M*_0_^1^ and *M*_0_^2^ will determine the volumes occupied by these phases, provided that the densities of these phases are the same or differ slightly. Therefore, by measuring the recovery of magnetization *M*_z_ as a function of time that has elapsed since the sample was amorphized, we obtain information on the ratios of *M*_0_^1^ and *M*_0_^2^ amplitudes. By further plotting the dependence of the crystalline phase amplitude on that time (after amorphization), the recrystallization rate can be determined. Fig. 11 shows the normalized value of *M*_0_^2^ component, which characterizes the crystalline phase in the system studied, depending on the time elapsed since sample amorphization. The recrystallization process involves gradual ordering of the side chains. One can suppose that it is related to the reconstruction of the bifurcated hydrogen bond network in crystalline sample.^14^

Bi-exponential recovery of magnetization was previously observed in cyclodextrin with diazepam,^48^ drugs in silica matrices,^49–51^ drugs with polymers,^23,52^ or in the process of recrystallization of FLD of different grain sizes^42^ of amorphous API.^53^

The measurement 14 weeks after amorphization showed a one-exponential magnetization recovery in all temperature range. This means that the sample has completely recrystallized and has become a single-phase system.

### 3.4. Molecular reorientations in a partially and fully recrystallized sample

For partially and fully recrystallized felodipine, the ^13^C CP MAS NMR spectrum was also recorded. The spectrum of the completely recrystallized sample corresponds to that of the crystalline sample, while the spectrum for the partially recrystallized FLD is definitely different. From the measurements of ^1^H *T*_1_ relaxation times carried out earlier, the degree of recrystallization was determined and is approximately 50%. All lines for this sample (in the range of 0–70 ppm) are narrower than for the amorphous sample, but only the lines corresponding to the carbon atom of the methyl group IV and the carbon atom in position 4 are single. In contrast, the lines corresponding to the carbon atoms of the methyl groups I and II (∼20 ppm) and groups III (∼50 ppm) are at least partially separated. The chemical shift indicates that the narrower line with a maximum at 50 ppm corresponds to the crystalline form, whereas the chemical shift of the wider line has a maximum at 51.9 ppm and is greater than the chemical shift for the amorphous form, which is equal to 50.9 ppm. The splitting of the lines suggests that there is a static disorder caused by the two positions of the carbon belonging to the methyl group III (by rotation around the C_15_–O_15b_ bond); one of these positions (at 50 ppm) corresponds to the crystalline form. The line corresponding to the carbon of the CH_2_ group is split into three lines. The first, at 60.4, corresponds to an amorphous form, while the line at 58.4 ppm corresponds to a crystalline sample. The third line, at 59.2 ppm, indicates, as above, the static disorder of the CH_2_ group. In summary, the analysis of ^13^C CP MAS spectra shows that the sample being studied is a mixture of amorphous and crystalline forms, with partially disordered side chains. As with the amorphous sample, *T*_1_ relaxation time measurements were made for individual carbon atoms. Two-exponential magnetization recovery was observed for all lines except that of the carbon atom belonging to methyl group IV. Relaxation times and magnetization amplitudes (*M*_0_^1^ and *M*_0_^2^) were determined using formulas (7)–(9). The results are shown in Table 3. The values of relaxation times correspond to the amorphous and crystalline samples, respectively, and their amplitudes are close to 50%, which confirm the sample was half-recrystallized. As the relaxation time for the C_19_ is one-exponential and its value is comparable with the crystalline FLD, in the partially crystallized sample, the reorientation of ethyl fragment around the O_16b_–C_18_ axis is activated.

**Table 3:** The activation energies for molecular reorientations in crystalline,^13^ amorphous and fully recrystallized FLD

	Crystalline^13^	Amorphous	Fully recrystallized
	*E*_a_ [kJ mol^−1^]	*E*_a_ [kJ mol^−1^]	*E*_a_ [kJ mol^−1^]
High-temp. min. (methyl no. IV)	13.76	7.8	14.4
Shallow – ethyl ester chain	9.5	—	8.3
Low-temp. min. (methyl no. I, II)	3.75; 0.74	6.8	4.18; 2.56
		5.4	
		4.4	
Low-temp. min. (methyl no. III)	4.18; 2.56	3.7	4.75; 0.61
		3.0	

**Fig. 12:**
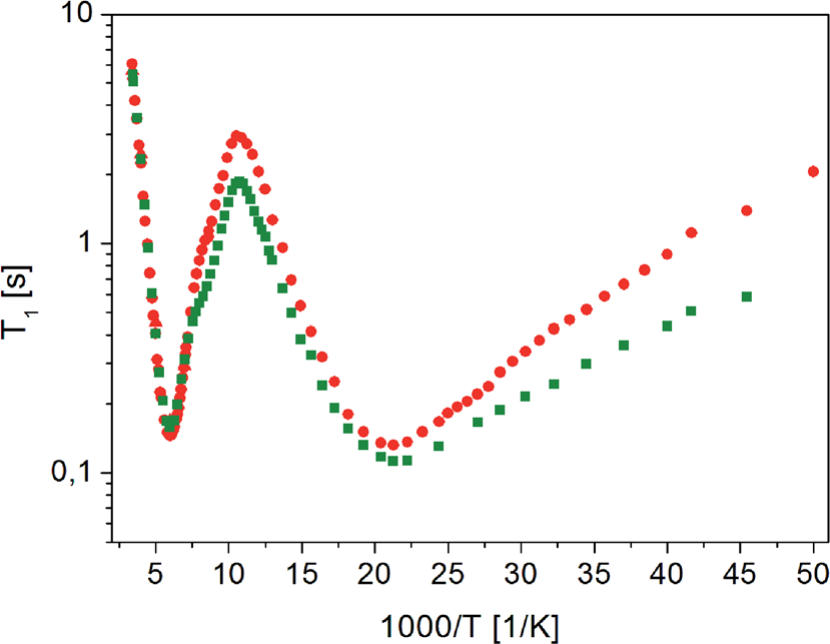
Dependence of relaxation time *T*_1_ on the reciprocal temperature for (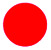) crystalline^13^ and (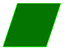) fully recrystallized FLD.

Measurements of ^1^H NMR relaxation times for the partially recrystallized sample showed that the temperature dependence of the *T*_1_^1^ relaxation time component covers the *T*_1_ relaxation time values for the amorphous sample, while the temperature dependence of the *T*_1_^2^ relaxation times is very similar to the dependence of *T*_1_ relaxation time for a crystalline sample, although the *T*_1_^2^ time values are slightly shorter than the *T*_1_ value for the crystalline sample. *T*_1_ measurements in the temperature range from room to 20 K in the fully recrystallized sample are shown in Fig. 12. All previously observed molecular reorientations for the crystalline sample, *i.e.* methyl groups and a fragment of a longer chain around the axis determined by the C_18_–O_16b_ bond, were identified in it. The activation parameters obtained are shown in Table 2.

As the analysis of the data contained in Table 2 shows, the activation parameters for the crystalline and recrystallized sample are very similar. However, small differences indicate that there was still some disorder in the sample and that the long-range ordering was not fully restored.

## 4 Conclusions

The microscopic model of molecular reorientation in amorphous, partially and fully recrystallized FLD was developed on the basis of ^1^H and ^13^C NMR and QENS studies.

Complete recrystallization of the bulk amorphous FLD sample occurs after 105 days. In the fully recrystallized sample, the same molecular reorientation as occurring in the crystalline form were identified, so all methyl groups reorientations and ethyl ester fragment. However, the values of activation parameters differ slightly from those observed for the crystalline form. This demonstrates that in the fully recrystallized FLD, the arrangement of the side chains is not exactly the same as in a crystalline form. In the partially recrystallized sample, static disorder caused by the two positions of both side chains was observed.

In amorphous FLD (stored at room temperature, in 0% RH), reorientation of all methyl groups was also detected, with the distribution of energy barrier and correlation times for groups I–III. Barrier heights for these reorientations are higher compared to those observed for the crystalline form. In contrast, methyl group IV is reoriented through a lower energy barrier compared to the crystalline form. In the amorphous form, at room temperature, correlation times characterizing the reorientations of methyl groups are in the range from 1 × 10^−10^ to 1 × 10^−12^ s. Both side chains are static disordered. The methyl and ethyl groups located at the ends of these chains occupy at least two positions, which are formed by rotation around bonds C_15_–O_15b_ and C_16_–O_16b_. In contrast to the crystalline form, in the amorphous form, no reorientation jumps of the ethyl ester fragment around the axis determined by the O_16b_–C_18_ bond. Inhibition of these reorientations is probably the reason for the reduction in the energy barrier height for reorientation of methyl group IV. The performed study revealed that the dynamics of side chains has a major influence on recrystallization process of amorphous felodipine.

## Conflicts of interest

There are no conflicts to declare.

## Supplementary Material

RA-010-D0RA07266D-s001
