# Supplementary material for: Exploring molecular reorientations in amorphous and recrystallized felodipine at the microscopic level
Source: RSC Adv. 2020 Oct 8;10(61):37346–57. doi: 10.1039/d0ra07266d (PMC9057141; doi:10.1039/d0ra07266d)
Supplement: RA-010-D0RA07266D-s001 [file RA-010-D0RA07266D-s001.pdf]

Electronic Supplementary Material (ESI) for RSC Advances.
This journal is © The Royal Society of Chemistry 2020

Supplementary Information

Exploring Molecular Reorientations in Amorphous and Recrystallized Felodipine at the Microscopic Level

A.Pajzderska[1*], J. Jenczyk[2], J. P. Embs[3] J. Wąsicki[1,2]

[1] Faculty of Physics, Adam Mickiewicz University, Uniwersytetu Poznańskiego 2, 61-614 Poznań, Poland
[2] NanoBioMedical Centre, Adam Mickiewicz University, Wszechnicy Piastowskiej 3, 61-614 Poznań, Poland
[3] Laboratory for Neutron Scattering and Imaging, Paul Scherrer Institut, 5232 Villigen, Switzerland

[*]corresponding author

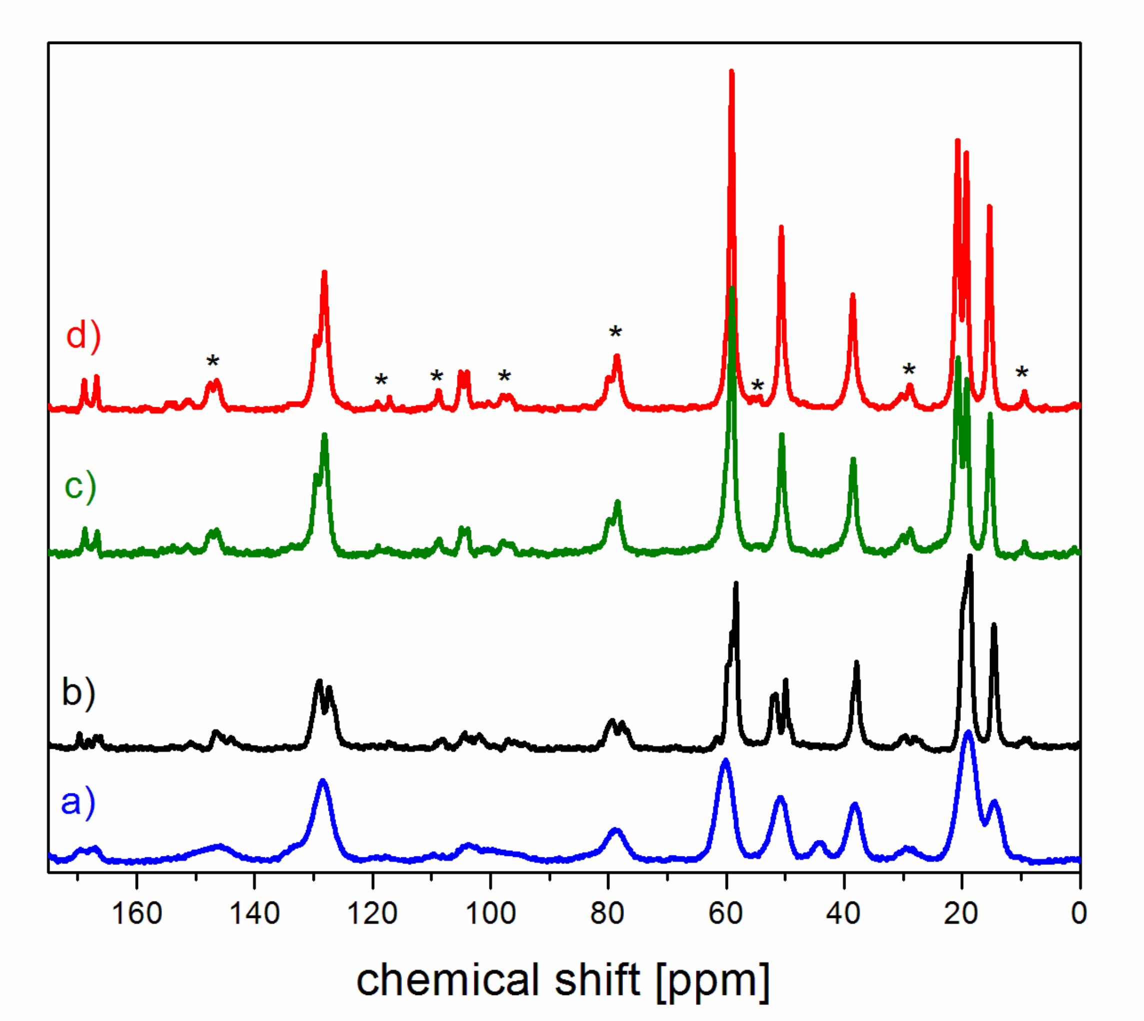

Fig. 1S. The ^{13}C CP MAS spectra of a) amorphous b) partially recrystallized c) fully recrystallized d) crystalline felodipine[1] The asterisks denote satellites.

Reference

1 A. Pajzderska, K. Drużbicki, M. A. Gonzalez, J. Jenczyk, J. Mielcarek, J. Wąsicki, Diversity of Methyl Group Dynamics in Felodipine: a DFT Supported NMR and Neutron Scattering Study, *Crystal Engineering Communications*, **2018**, 20, 7371-7385.
